# The unpredictable nature of bubble evolution

**DOI:** 10.1038/s41598-022-23231-8

**Published:** 2022-12-01

**Authors:** Jack Lawless, Jack Keeler, Antoine Gaillard, Andrew Hazel, Anne Juel

**Affiliations:** 1 Manchester Centre for Nonlinear Dynamics, University of Manchester, Oxford Road, Manchester, M13 9PL UK; 2 School of Mathematics, University of East Anglia, Norwich Research Park, Norwich, NR4 7TJ UK; 3 Van der Waals-Zeeman Institute, University of Amsterdam, Science Park 904, Amsterdam, The Netherlands

**Keywords:** Fluid dynamics, Nonlinear phenomena

## Abstract

Unpredictable dynamics arising from a sensitivity to initial conditions is commonly associated with chaos. We demonstrate how similar unpredictability manifests in a nonlinear system that possesses a large number of long-term outcomes, namely the propagation of an air bubble within a viscous fluid-filled channel. The system under investigation supports various stable states of single-bubble propagation. In addition, bubbles can readily break up during their propagation. Upon subjecting steadily-propagating bubbles to finite-amplitude perturbations in the form of localised channel constrictions, we identify localised regions of the driving flow rate for which the resulting evolutions are unpredictable. Visibly-indistinguishable bubbles are observed to evolve towards a multitude of long-term outcomes, including each of the stable states available to the initial bubble and various states of permanently-changed bubble topology. By combining high-precision experimental results with simulations of a depth-averaged lubrication model of the system, we determine that this behaviour is driven by a sensitive dependence on initial conditions within the vicinity of an unstable periodic orbit.

## Introduction

Complex systems are constructed from various interacting subsystems; they are found ubiquitously in nature and often exhibit collective nonlinear behaviours that appear difficult to interpret from the outset^1^. However, there is potential for successful predictions to be made in systems as complex and chaotic as the climate, as recently highlighted by the award of the 2021 Nobel Prize in Physics “for groundbreaking contributions to our understanding of complex systems”^2^. A key ingredient is modern dynamical systems theory, which has recently enabled major advances in the understanding of the subcritical transition to turbulence in shear flows by providing the appropriate framework required to elucidate such behaviour^3^. The core element of this approach is to determine the set of invariant states that are contained within a system’s phase space. The fundamental reasoning behind this is that these states, depending on their stability, act locally to either attract or repel trajectories and, thus, they can be viewed as orchestrators of the system’s time-evolution.

**Figure 1:**
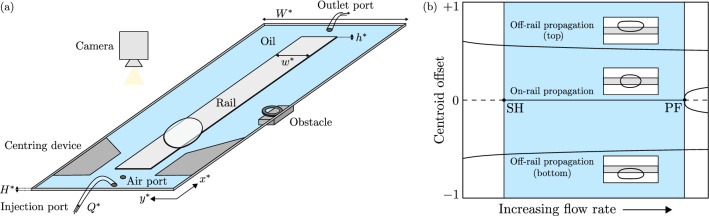
(**a**) Schematic view of the experimental Hele–Shaw channel, which features an axial-centred elevation, or rail, along its length. An obstacle is placed downstream of the centring device, in contact with one of the channel side-walls, resulting in a local constriction of the channel width. (**b**) Schematic of the system’s underlying steady state space with variation of the driving flow rate. Each state is characterised by the normalised lateral offset of the bubble centroid from the channel centreline. The shaded region of interest is bounded between a subcritical Hopf bifurcation (SH) and a supercritical pitchfork bifurcation (PF). Within this region, there are three stable states available to the initial bubble: steady on-rail propagation about the channel centreline and steady off-rail propagation, in which the lateral placement of the bubble is biased towards one of the channel side-walls. Snapshots of the states are shown in the associated insets.

Similar initial states of a time-evolving system need not necessarily converge towards the same long-term outcome. Visibly-indistinguishable initial states of a nonlinear system have the potential to exponentially diverge from one another and, consequently, consecutive evolutions of the system under the same conditions may result in profoundly different long-term outcomes. This behaviour, henceforth referred to as practical unpredictability, is commonly associated with chaos. Real-world systems such as the weather^4^, human economies^5^ and the spread of infectious diseases within a population^6^ readily exhibit unpredictable long-term dynamics, although many of these systems also exhibit stochasticity.  Furthermore, these systems are usually highly complex and evolve over a myriad of spatial and temporal scales. Nonetheless, practical unpredictability can occur in much simpler, deterministic systems. In this paper, we demonstrate how unpredictable dynamics manifest in a spatiotemporal system that does not exhibit chaos, neither transiently nor long-term, within the relevant parameter regime: the propagation of an air bubble within a viscous fluid-filled channel.

Fluid motion has long been a testbed for complex nonlinear behaviour. Turbulence, i.e. disorder in space and time, is a common state of large-scale flows^7^, whilst temporal disorder, or chaos, is routinely observed in spatially-confined flows^8^. In this paper, the fluids are confined to a channel much wider and longer than it is deep: a Hele–Shaw channel. The channel is partially occluded by a small elevation, henceforth referred to as a rail, that is applied along its axial centreline; see Fig. 1a. An air bubble is propagated through the channel by imposing a constant volume flux flow of oil; negligible inertial forces mean that nonlinearities arise exclusively at the air-oil interface and, hence, the dynamical state of the system is encoded in the shape of the bubble. Depending on the experimental parameters, the bubble is able to exhibit a rich variety of complex nonlinear behaviours, examples of which include multistability and time-periodic modes of propagation^9–11^. We focus our study on a range of driving flow rates for which there are three stable single-bubble states, consisting of  a steady ‘on-rail' state, in which the bubble propagates symmetrically about the channel centreline, and two steady ‘off-rail' states, in which the bubble propagates asymmetrically about the channel centreline. It is noted that the on-rail and off-rail states correspond to disconnected solution branches of the governing equations^10^. A simplified schematic of the system’s steady state structure is provided in Fig. 1b, whereas a more detailed version can be found in Keeler et al.^10^. In addition, bubbles also readily exhibit complex transient dynamics that can result in their breakup. The resulting post-breakup bubbles may or may not recombine during their subsequent evolution. This distinctive, yet rather peculiar, feature of the system allows for topological changes in its underlying phase space that are not described by conventional bifurcations. Bubble dynamics have previously been found to be well-modelled by a set of two-dimensional depth-averaged lubrication equations^9,12^, which are more amenable to numerical analysis than the three-dimensional Navier–Stokes equations. Hence, this system acts as a suitable toy model to investigate fundamental nonlinear behaviour that could not easily be isolated in systems of increased complexity.

We apply controlled, finite-amplitude perturbations to bubbles that initially propagate steadily on-rail. For fixed perturbations, we identify localised intervals of the driving flow rate for which the subsequent evolution of the system is unpredictable because visibly-indistinguishable bubbles evolve towards a multitude of distinct long-term outcomes following a common transient oscillatory response to their perturbation. The observed outcomes include each of the three stable single-bubble states, as well as various states of permanently-changed bubble topology following bubble breakup. We contrast our experimental observations with related numerical simulations and employ concepts from dynamical systems theory in order to interpret the localised occurrences of practical unpredictability in the experiment and their relation to the amplitude of perturbation.

**Figure 2:**
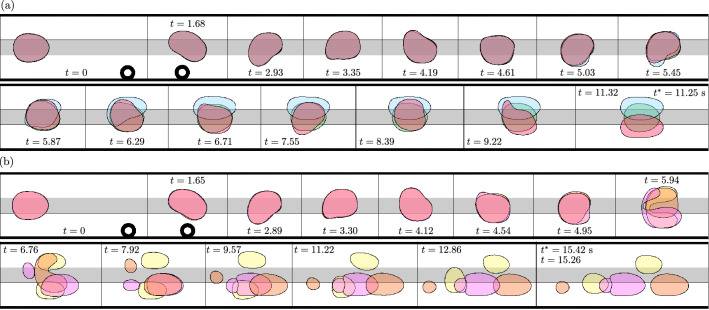
(**a**,**b**) Superimposed experimental time-sequences of bubble evolutions following their circumvention of an obstacle, which constricts the channel width by $21.7\%$. In each case, the dimensionless flow rate is $Q=0.018$. The bubble centroids are aligned in the streamwise direction. The time-sequences in (**a**) are examples of direct evolutions towards each of the three stable states available to the initial bubble, whereas the time-sequences in (**b**) are examples of evolutions that feature bubble breakup.

Interpreting the behaviour of a system in response to an external perturbation necessitates the study of its transient dynamics. Although they are never observed explicitly in experiments, unstable invariant states are able to influence a system’s transient dynamics significantly^13^. In particular, weakly-unstable invariant states, commonly referred to as saddles, are able to orchestrate complex transient dynamics within the vicinity of their locally-attracting stable manifolds^14,15^. This could, for instance, lead to transient chaos,  in which a system exhibits chaotic dynamics as it passes through certain regions of its phase space, before ultimately settling in a stable long-term outcome^16,17^. The influence of weakly-unstable states on the subcritical transition to turbulence in linearly stable shear flows has also been recognised in a plethora of recent studies, widely formalising the notion of so-called ‘edge states’ of a dynamical system^3,18–24^. An edge state is defined as a weakly-unstable invariant state, whose stable manifold forms part of the basin boundary that separates two distinct long-term dynamical outcomes. Two commonly encountered classes of edge states are saddle equilibria and unstable periodic orbits (UPOs), the latter of which bear a strong significance in modern theories of chaos and turbulence^25–31^. In fact, Keeler et al.^10^ employed edge-tracking techniques to reveal the existence of an isolated UPO in the depth-averaged model of our system, emanating from a subcritical Hopf bifurcation. In this paper, we demonstrate how the influence of an isolated UPO, when combined with a large number of potential long-term outcomes, is sufficient to drive long-term practical unpredictability in a real-world system in the absence of any form of chaotic dynamics.

## Results

### Experimental results—small perturbation

The experiments that were performed in this study are conceptually simple: bubbles of fixed size were generated and subsequently propagated steadily on-rail towards  a circular obstacle placed downstream. The relevant control parameters are given by the dimensionless flow rate *Q* and the obstacle size, which is parameterised in terms of the percentage ratio between its diameter and the width of the channel. We investigate the evolution of the system following the obstacle-induced deformation of a bubble. Bubble evolutions were filmed in top-view by a steadily translating camera and, in this paper, they are represented by a series of instantaneous snapshots of the bubble contour. Time-labels are given in terms of a dimensionless time *t*. A comprehensive description of the experimental setup and protocols used to reproducibly generate and propagate bubbles of prescribed size, alongside details pertaining to the nondimensionalisation process, are provided in “Materials and methods”.

**Figure 3:**
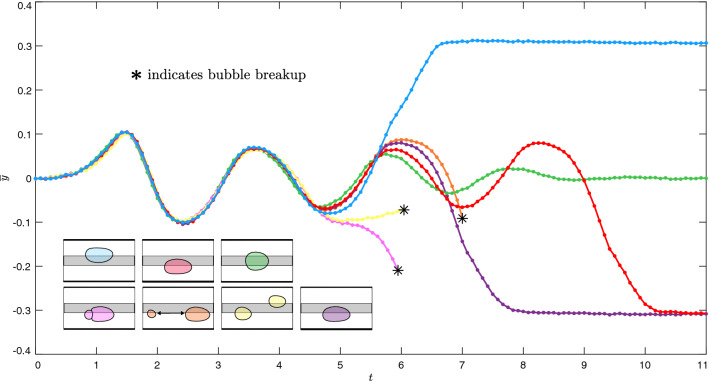
Time-evolution of the bubble centroid lateral offset, denoted $\overline{y}$, for each bubble in Fig. 2a,b and an additional (purple-coloured) bubble which evolves towards off-rail propagation on the bottom of the rail. The first peak and trough of each curve correspond to the bubble’s circumvention of the obstacle. Insets: final states of the bubbles.

We begin with the experimental observations following the perturbation of a bubble through the use of a modestly-sized obstacle that constricted the channel width by $21.7 \%$. Within a small interval of the dimensionless driving flow rate ($0.018< Q < 0.019$), visibly-indistinguishable bubbles were observed to evolve towards multiple distinct long-term outcomes at the same experimental parameters following their circumvention of the obstacle. A set of three experimental time-sequences are superimposed in Fig. 2a where, in each case, a bubble is propagated towards the obstacle at $Q=0.018$. The bubbles deform significantly as they circumvent the obstacle at $t=1.68$. Each bubble begins to oscillate transiently as it propagates away from the obstacle. At early times during their oscillation, e.g. at $t=2.93$ and $t=3.35$, the bubbles remain indistinguishable. However, small-scale deviations in the shapes of their interfaces become visibly apparent by $t=4.19$, which grow significantly by $t=5.45$. In each case, the oscillations are not sustained and each bubble proceeds to evolve directly towards one of the three single-bubble states at some later time.

In Fig. 2b, an additional set of three experimental time-sequences are superimposed. In each case, the early-time transient dynamics are the same as described previously. However, each bubble proceeds to break up at some point during its oscillation. The resulting pairs of post-breakup bubbles are distinct in each case and evolve towards profoundly different long-term outcomes. The magenta-coloured bubbles settle on the same side of the rail and aggregate to form an off-rail compound bubble. As described in Gaillard et al.^11^, compound bubbles ultimately coalesce to form a simple bubble once the oil layer separating them has drained and, hence, this particular evolution can be viewed as a transient excursion towards a stable off-rail state. The orange-coloured post-breakup bubbles also settle on the same side of the rail. However, instead of aggregating, the two bubbles separate indefinitely and form a two-bubble state of permanently-changed topology. Finally, the yellow-coloured post-breakup bubbles also indefinitely separate, albeit on opposite sides of the rail. For each bubble shown in Fig. 2a,b, we plot the time-evolution of the lateral offset of its centroid from the channel centreline, denoted $\overline{y}$, in Fig. 3. Here, the divergence of the bubbles is demonstrated explicitly.

### Numerical results—small perturbation

The experimental observations are consistent with the transient exploration of a UPO by the bubbles following their perturbation. As mentioned previously, Keeler et al.^10^ determined the existence of a UPO in the depth-averaged model, emanating from a subcritical Hopf bifurcation. In this section, we compare the experimental results with simulations of the model. Descriptions of both the model’s implementation and the numerical methods used to solve the resulting equations are provided in “Materials and methods”.

**Figure 4:**
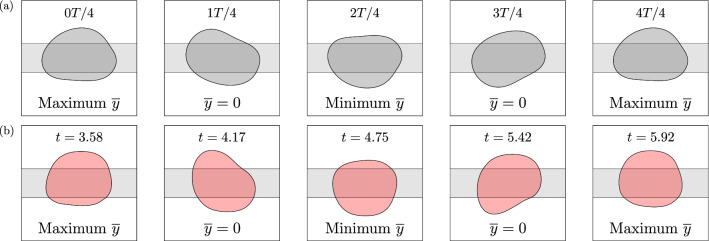
(**a**) Simulated time-sequence of the UPO dynamics for a bubble of size $r=0.54$ at dimensionless flow rate $Q=0.032$ in the depth-averaged model. The bubble is shown at equally-spaced time intervals of $t=T/4$, where *T* is the computed period of the UPO. (**b**) Experimental time-sequence of the transient oscillations that were observed experimentally. The data is taken from the red-coloured bubble in Fig. 2a.

We simulated the time-evolution of bubbles initially propagating steadily on-rail after applying linear perturbations with the UPO eigenmode; refer to Eq. (8) in “Materials and methods”. The phase of perturbation was fixed arbitrarily at $\varphi = 6\pi / 12$. We note that there is a known discrepancy between the value of the subcritical Hopf point, denoted $Q_H$, in the experiments and the model; this is detailed in Gaillard et al.^11^. Hence, the simulated flow rate was chosen such that the absolute distance from the subcritical Hopf point ($Q-Q_H$) was the same as in the experiments. Convergence towards the UPO was achieved via an edge-tracking procedure, in which the perturbation strength $\varepsilon$ was varied through interval bisection. We let $\varepsilon _U$ denote the value of the perturbation strength that resulted in a sufficient convergence towards the influencing vicinity of the UPO; refer to Eq. (8) in “Materials and methods”. Figure 4a shows a time-sequence of the simulated UPO dynamics for a bubble whose size is equal to that of the experimental bubbles. We compare the simulation directly to the mode of oscillation observed in the experiments in Fig. 4b. In general, there is an excellent qualitative agreement between the two. It is noted that the mirror-symmetry of the experimental bubble at particular points during its evolution, e.g. in the second and fourth panels of Fig. 4b, is also consistent with the assumed symmetry of such an orbit due to the symmetry of the flow domain in the *y*-direction. The evidence suggests that the bubbles were indeed perturbed within the influencing vicinity of the UPO in the experiments and, hence, that their transient evolutions correspond to its exploration.

**Figure 5:**
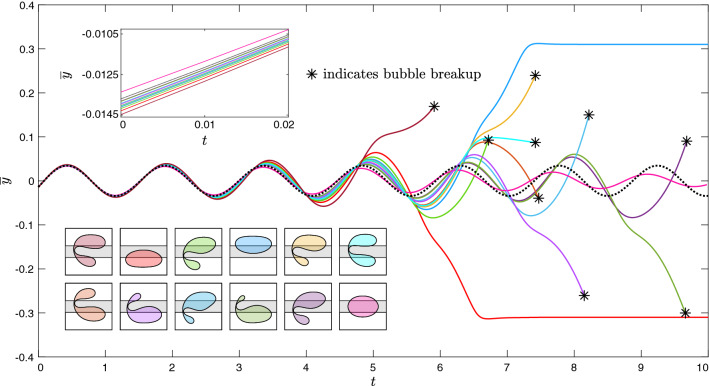
Time-evolutions of the bubble centroid lateral offset $\overline{y}$ following a series of marginally-different initial perturbations applied to bubbles of size $r=0.54$ at dimensionless flow rate $Q=0.032$ in the depth-averaged model. The projection of the UPO in this space is represented by the black dashed curve. Bottom inset: bubble shapes at the final time-step, where the colour of each bubble corresponds to the same colour curve. Top inset: zoom-in on the initial values of $\overline{y}$ and after the first two time-steps.

In an attempt to replicate the observed experimental unpredictability, we then simulated bubble evolutions for marginally-different values of the perturbation strength close to $\varepsilon _U$, the results of which are shown in Fig. 5. For each bubble, we show the time-evolution of its centroid’s lateral offset $\overline{y}$, in addition to a snapshot of its shape at the final time-step. It is noted that simulations were terminated either when the bubble reached a stable steady state or at the point of bubble breakup. The projection of the UPO in this reduced space is denoted by the black dashed curve. The curves begin almost superposed at $t=0$, with initial differences less than $0.2\%$ of the characteristic length-scale ($W^*/2$). Each bubble proceeds to transiently explore the UPO, completing several cycles of oscillation with near-constant amplitude. Depending on the value of $\varepsilon$, divergence from the UPO occurs either through the monotonic decay or the monotonic growth of the oscillation amplitude. For $\varepsilon <\varepsilon _U$, the amplitude of oscillation decays and bubbles return to steady on-rail propagation. For $\varepsilon >\varepsilon _U$, the amplitude of oscillation grows and bubbles either evolve directly towards a state of steady off-rail propagation or break up. In the latter case, various unique pairs of post-breakup bubbles are formed. Extreme examples of breakup include the cyan-coloured bubble, which breaks near-symmetrically, and the dark green-coloured bubble, which breaks strongly asymmetrically. Several intermediate examples of breakup are also shown. Based on these results, we infer that a continuum of size fractions between the resulting post-breakup bubbles is possible. Although we do not pursue the resulting post-breakup dynamics in this paper, based on a previous study of two-bubble dynamics by Keeler et al.^32^, it is expected that the simulations would capture the added multiplicity of long-term behaviours that were observed experimentally.

**Figure 6:**
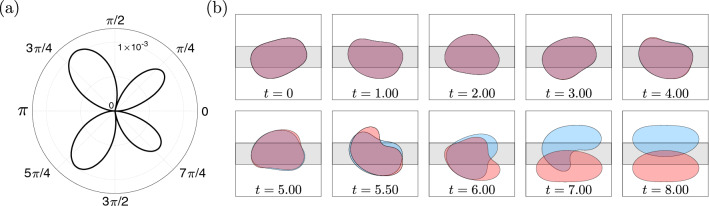
(**a**) Dimensionless radial difference between the initial bubble shapes at angular position $\theta$, as measured from the axial centreline of the channel, for the two bubbles which evolve towards steady asymmetric propagation on either side of the rail in Fig. 5. (**b**) The corresponding time-sequences of their evolutions.

In Fig. 6a, the dimensionless radial difference between the initial shapes of the two bubbles that later evolve towards steady off-rail propagation on opposite sides of the rail in Fig. 5 is plotted as a function of the angular position $\theta$, as measured from the axial centreline of the channel. The initial deviations at fixed $\theta$ are less than $0.2\%$ of the characteristic length-scale and, hence, the two bubbles appear visibly indistinguishable. In Fig. 6b, we superimpose the simulated evolutions of the two bubbles; here it can be seen that much of the characteristic behaviour that was observed experimentally also transpires in the model. This includes a close alignment of their interfaces early during their evolutions, followed by the emergence of small-scale deviations that later grow in amplitude. By defining suitable metrics of separation between neighbouring bubble trajectories, e.g. the absolute difference between $\overline{y}$ and similar integral measures of the difference between their shapes, it was determined that this behaviour arises a direct result of the exponential separation of neighbouring trajectories in the vicinity of the UPO. Refer to Supplementary Fig. S1 for more details. In "Mechanisms of unpredictability" section, the underlying mechanisms that lead to this behaviour are discussed.

**Figure 7:**
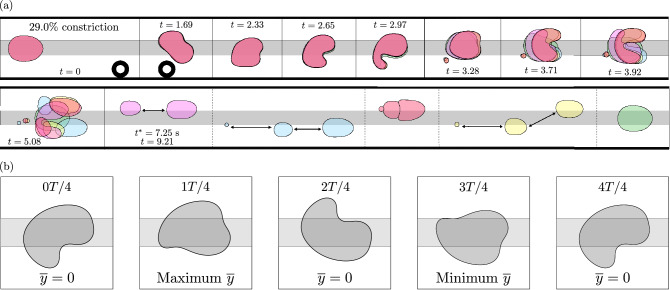
(**a**) Superimposed experimental time-sequences of bubble evolutions resulting from the circumvention of a larger obstacle that constricted the channel width by $29.0\%$. In each case, the dimensionless flow rate is $Q=0.023$. The bubble centroids are aligned in the streamwise direction. Arrows indicate long-term separation between bubbles. We note that snapshots of the final states are not superimposed, nor do they represent the relative position of each bubble in the channel. (**b**) Simulated time-sequence of the UPO dynamics for a bubble of size $r=0.54$ at dimensionless flow rate $Q=0.037$ in the depth-averaged model. The bubble is shown at equally-spaced time intervals of $t=T/4$, where *T* is the computed period of the UPO.

### Experimental and numerical results - larger perturbations

Upon increasing the amplitude of the applied perturbation through the use of a larger obstacle, which constricted the channel width by $29.0\%$, a localised region of unpredictability was identified for $0.023< Q < 0.024$. In Fig. 7a, we present a set of superimposed experimental time-sequences where, in each case, a bubble is propagated towards the obstacle at $Q=0.023$. The onset of transient oscillations again occurs following circumvention of the obstacle at $t=1.69$ and the dynamics are found to exhibit an excellent qualitative agreement with those of the UPO at the same value of $(Q-Q_H)$ in the depth-averaged model, as demonstrated in Fig. 7b. Notably, the ‘tail-like’ shape of the bubble rear at $t=2.65$ and $t=3.71$ align closely with the simulations at 0*T*/4 and 2*T*/4, respectively. In both experiments and simulations, the period of oscillation is determined to remain unchanged from that at the lower flow rate; refer to Supplementary Fig. S2 for more details. Importantly, this indicates that the UPO has not bifurcated. It is known that bifurcations of UPOs can result in complex dynamics. For example, period-doubling bifurcations can lead to chaos. However, there are no signs of this occurring and, hence, the observed oscillations are inferred to be an exploration of the same, albeit increased amplitude, UPO. Relative to those observed at the lower flow rate, the oscillations are short-lived. Namely, in each case, the bubble completes less than a single cycle of oscillation. The bubbles also deform more significantly, resulting in more frequent breakups. Direct evolutions towards either of the stable off-rail steady states were never observed and, instead, only occurred indirectly as a result of aggregation and coalescence following bubble breakup. Secondary breakups of post-breakup bubbles were also observed frequently, leading to more exotic long-term states of permanently-changed topology. For instance, the blue-coloured and yellow-coloured bubbles in Fig. 7a evolve towards long-term three-bubble states.

For the most extreme constriction investigated, which constricted the channel width by $33.9\%$, unpredictable dynamics did not arise at any flow rate within the region of interest. Transient oscillations were never observed and bubble evolutions were found to be entirely reproducible upon a succession of repeat experiments at the same experimental parameters. For most flow rates within the range of interest, bubbles directly evolved towards steady off-rail propagation on the side of the rail opposite to the placement of the obstacle following their circumvention of the obstacle. For sufficiently high flow rates, bubbles were instead observed to break up following their circumvention of the obstacle. The occurrence of breakup may be attributed in part to the decreasing influence of surface tension.

## Discussion

### Mechanisms of unpredictability

Based on the established similarities between the experimental and simulated dynamics, we infer that practical unpredictability arises in the experiments as a direct result of the exponential separation of neighbouring trajectories in the vicinity of the UPO. In this section, we discuss the underlying mechanisms that lead to this behaviour.

**Figure 8:**
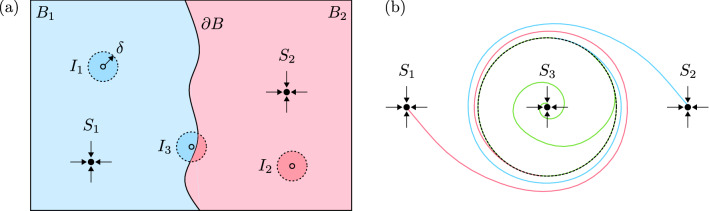
(**a**) Two-dimensional schematic, demonstrating how unpredictability can arise at a smooth basin boundary $\partial B$. The basins of attraction, denoted by $B_1$ and $B_2$, are the sets of initial conditions that evolve towards the fixed point attractors $S_1$ and $S_2$, respectively. The initial conditions $I_1$, $I_2$ and $I_3$ are uncertain within precision $\delta$. (**b**) Two-dimensional schematic demonstrating how a UPO (denoted by the black dashed circle), in the presence of multiple co-existing attractors, can give rise to unpredictability. Two attractors, $S_1$ and $S_2$, lie within the exterior region and a single attractor, $S_3$, lies within the interior region. Three arbitrary trajectories emanate from the UPO, each of which evolve towards one of the attractors.

The long-term outcome of any initial state of a dynamical system containing multiple co-existing attractors is determined solely by the basin of attraction within which the state lies. In the immediate vicinity of a basin boundary, long-term unpredictability can manifest in practice as a result of the inherent uncertainty that accompanies any initial state^33–35^. The model two-dimensional phase space in Fig. 8a acts to demonstrate this concept. Two fixed point attractors, denoted $S_1$ and $S_2$, reside within the model phase space and their associated basins of attraction, denoted $B_1$ and $B_2$, are separated by the smooth, common basin boundary $\partial B$. Three arbitrary initial states of the system, labelled $I_1$, $I_2$ and $I_3$, are surrounded by disks of small, yet finite radius $\delta$, representing the inherent uncertainty that accompanies their experimental measurement. The entirety of the disks of uncertainty within which $I_1$ and $I_2$ lie are contained within a single basin of attraction, meaning that these two initial states predictably evolve towards $S_1$ and $S_2$, respectively. However, the disk of uncertainty within which $I_3$ lies simultaneously overlaps both $B_1$ and $B_2$, meaning that there is a non-zero probability of the initial state evolving towards either $S_1$ or $S_2$. Hence, its long-term outcome is rendered unpredictable.

Figure 8b demonstrates a related, two-dimensional model of a route to unpredictability that arises if multiple attractors co-exist in either of the two regions of phase space that are separated by the basin boundary formed by a UPO. Three fixed-point attractors reside within the phase space; both $S_1$ and $S_2$ are situated within the ‘exterior’ region, whilst $S_3$ is situated within the ‘interior’ region. Because there is only a single attractor contained within the interior region, all trajectories that emanate inwards from the UPO must evolve towards $S_3$. Trajectories that emanate outwards from the UPO must evolve towards either $S_1$ or $S_2$; this leads to the formation of finely-structured, alternating ‘mosquito coil’-shaped basins of attraction that originate and spiral outwards from the UPO^34^. As the phase space separation from the UPO decreases, so must the ‘width’ of the alternating coils. Beyond a sufficiently small separation, the width of the coils becomes less than that of the system’s intrinsic uncertainty. Consequently, it becomes possible for visibly-indistinguishable states situated sufficiently close to the UPO to evolve towards any of the three attractors as a result of the aforementioned unpredictability within the vicinity of a basin boundary. It is remarked that this formalism is generic and, hence, can be extended to an arbitrary number of both internal and external attractors.

**Figure 9:**
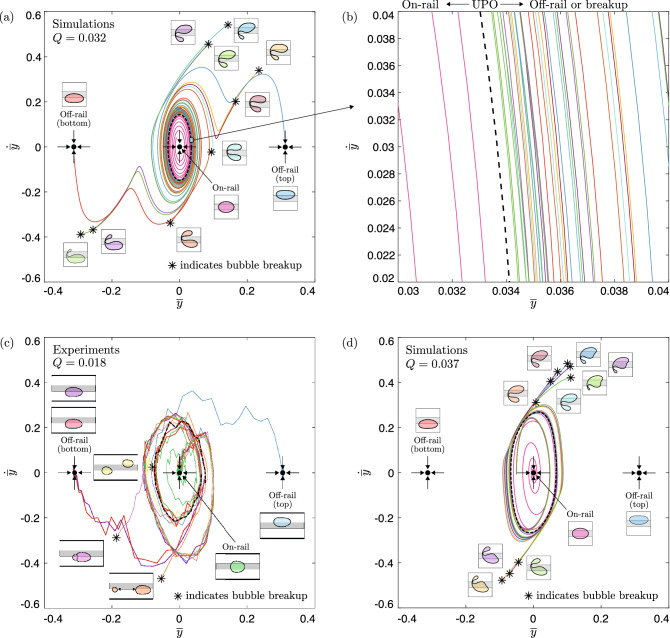
(**a**) Projection of the numerically-computed trajectories in the $(\overline{y}, \, \dot{\overline{y}})$ plane at $Q=0.032$. The UPO is indicated by the black dashed trajectory. (**b**) Close-up of the highlighted region, emphasising the spiralling nature of the outwardly-emanating trajectories in the vicinity of the UPO. (**c**) Projection of the experimental trajectories in the $(\overline{y}, \, \dot{\overline{y}})$ plane at $Q=0.018$. The black dashed trajectory corresponds to the first cycle of oscillation completed by the red-coloured bubble. (**d**) Projection of the numerically-computed trajectories in the the $(\overline{y}, \, \dot{\overline{y}})$ plane at $Q=0.037$.

In Fig. 9a, the simulated bubble trajectories of Fig. 5 are projected onto the $(\overline{y}, \, \dot{\overline{y}})$ plane; it is noted that the apparent intersection of trajectories occurs due to the projection of a higher-dimensional space onto a reduced-dimensional space. The structure of the system’s phase space in this projection is qualitatively consistent with the two-dimensional schematic outlined in Fig. 8b. Here, the on-rail state and two off-rail states are analogous to the interior and two exterior attractors, respectively. However, in addition to the two exterior off-rail states, the propensity of a bubble to break up provides the system with a continuum of additional exterior breakup states. This means that the set of outwardly-emanating trajectories encompasses, effectively, an infinite number of potential long-term outcomes. The spiralling of the system’s trajectories, as further highlighted in Fig. 9b, provides a sensitive dependence on initial conditions in the vicinity of the UPO and, hence, allows for the evolution of visibly-identical bubbles towards any of these potential long-term outcomes. An analogous phase space projection of the experimental trajectories of Fig. 3 is provided in Fig. 9c, where many qualitative similarities to its simulated counterpart can be observed. The simulated phase space projection is found to remain qualitatively unchanged at the higher flow rate investigated; see Fig. 9d.

### Conclusion

**Figure 10:**
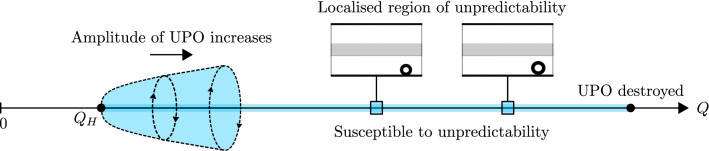
Schematic of the region of parameter space that is susceptible to unpredictable dynamics; this region persists from the birth of the UPO at $Q_H$ until the UPO is destroyed. As *Q* increases beyond $Q_H$, larger finite-amplitude perturbations are required in order to provoke unpredictability due to the growing amplitude of the UPO. For a fixed perturbation, unpredictable dynamics transpire within a localised region of *Q*.

In this paper, we have showcased how unpredictable dynamics can arise in the two-phase flow of a bubble propagated within a viscous fluid-filled Hele–Shaw channel. This complex phenomenon was elucidated by employing concepts from modern dynamical systems theory. In particular, synthesis of experimental results with related simulations of a depth-averaged lubrication model of the system allowed us to determine that the localised regions of unpredictability that were observed in experiments are driven by a sensitive dependence on initial conditions within the vicinity of a UPO. The ability of a bubble to break up was found to further enhance its extent of unpredictability, with both experiments and simulations indicating that there are, effectively, an infinite number of potential long-term outcomes for a bubble perturbed within its vicinity. This unique attribute of the system distinguishes it from other systems that exhibit unpredictability in the form of a small number of potential long-term outcomes and, in fact, may also be a key reason as to why unpredictability is observed so readily in the experiments.

Although unpredictable dynamics are confined to a small, localised region of flow rate for a fixed perturbation in the experiments, the underlying mechanism is generic. Hence, we expect that unpredictability will be present throughout the entirety of the range of flow rates for which the UPO exists, provided that the system is perturbed appropriately. As such, a large proportion of its parameter space becomes susceptible to unpredictability and this is demonstrated schematically in Fig. 10. It is remarkable that this pervasive manifestation of unpredictability can be generated by the presence of a single UPO in a parameter regime where the system is otherwise ordered. We expect that the findings of this paper can be extended generally to other nonlinear systems.

## Materials and methods

### Experimental methods

The experiments throughout this paper were performed in the same Hele-Shaw channel as described in Gaillard et al.^11^, where a comprehensive description of both the experimental setup and bubble-generation procedure can be found. A schematic diagram of the setup is shown in Fig. 1a, for which we will outline the important features. The channel consisted of two horizontally-levelled float-glass plates, separated by two parallel strips of stainless steel shim which were bonded to the bottom glass plate. The thickness of these steel shims was $H^* ={1.00} \pm 0.001\,\hbox { mm}$ and they were separated by a distance $W^* = {40.0} \pm 0.1\,\hbox { mm}$, resulting in a cross-sectional aspect ratio $\alpha = W^*/H^* = 40$. An axially-centred, uniform depth-perturbation, henceforth referred to as a ‘rail’, was added to the bottom glass plate in the form of a strip of translucent adhesive tape. The rail had width $w^* = {10.0} \pm 0.1\,\hbox { mm}$, thickness $h^* ={24} \pm 1\,\upmu \hbox {m}$ and spanned the entirety of the channel length.

The channel was filled with silicone oil (Basildon Chemicals Ltd) of dynamic viscosity $\mu = {0.019}\,\hbox {Pa\,s}$, density $\rho = {951}\,\hbox {kg}\,\hbox {m}^{-3}$ and surface tension $\sigma = {21}\,\hbox {mN}\,\hbox {m}^{-1}$. Experiments were performed at the ambient laboratory temperature of ${21}{\pm }1\,^{\circ }\hbox {C}$. The flow of silicone oil was controlled via a network of syringe pumps and solenoid valves; a three-way solenoid valve was used to link the network of injection syringes, the channel inlet and the oil reservoir and a two-way solenoid valve was used to link the channel outlet and the oil reservoir. Depending on the configuration of the valves, oil could be injected into or withdrawn from the channel at a constant volumetric flow rate $Q^*$ or withdrawn from the oil reservoir to fill the injection syringes. Air bubbles were generated by opening an air valve situated a short distance downstream of the channel inlet, whilst slowly withdrawing a prescribed volume of oil at the channel outlet through a syringe.

Following their detachment from the air injection port, bubbles were initially centred on the rail by their propagation through a centring device, consisting of a localised symmetric channel constriction followed by a linear expansion region. They were then propagated through the channel by imposing a constant dimensionless driving flow rate $Q=\mu U_0^* / \sigma$, where $U_0^* = Q^* / W^* H^*$ is the average velocity of oil in an equivalent unperturbed channel. The bubbles were filmed in top-view by a CMOS camera mounted onto a motorised translation stage, which moved at a constant velocity based on an empirical relationship between the bubble propagation velocity and the driving flow rate as determined in Gaillard et al.^11^, ensuring that the bubbles remained in the field of view of the camera for the duration of the experiment. Control of the syringe pumps, valves and camera were interfaced to a computer and controlled via a custom-built LabVIEW code.

In order to perturb a steadily-propagating bubble, a circular steel washer, henceforth referred to as an ‘obstacle’, was placed in contact with one of the channel side-walls and fixed in place by a N52-grade neodymium magnet situated below the bottom glass plate. The three obstacle sizes which we used constricted the width of the channel by a maximum of $21.7\%$, $29.0\%$ and $33.9\%$, as measured along their circumferences.

The raw data obtained by the camera was treated by a series of image-processing filters and bubble contours were identified through the application of a Canny edge-detection algorithm. We present all experimental figures in a frame of reference moving with the bubble centroid. Length scales are non-dimensionalised by the channel half-width $W^*/2$ in the ‘in-plane’ directions and by the channel height $H^*$ in the transverse direction. We parameterise bubble size by defining a non-dimensional radius $r=2r^*/W^*$, where $r^* = \sqrt{A^*/\pi }$ and $A^*$ is the projected area of the bubble. Throughout this paper, the bubble size is fixed at $r=0.54$. We choose the characteristic time-scale $t=2U_0^* t^* / W^*$, based on the average time taken for oil to propagate a distance equal to one channel half-width at flow rate $Q^*$ in an equivalent unperturbed channel.

### Numerical methods

Simulations of bubble propagation were carried out using a depth-averaged lubrication model, as detailed in Thompson et al.^12^. The validity of this model in describing the dynamics of single air bubbles has been verified in Franco-Gomez et al.^9,36^ and Keeler et al.^10^. Multiple-bubble interactions have also recently been studied in both Gaillard et al.^11^ and Keeler et al.^32^. Here, we recall the important details.  We implement the same nondimensionalisation procedure as outlined above. The rail is modelled by a piecewise-smooth, axial-centred channel depth profile of the form

1 $$\begin{aligned} b(y) = 1-\frac{h}{2} \bigg [ \tanh {\big (s(y+w) \big )} - \tanh {\big (s(y-w) \big )} \bigg ], \end{aligned}$$

where $h=0.024$ and $w=0.25$ are the dimensionless height and width of the rail, respectively, and $s=40$ is a parameter which controls the sharpness of the rail edges, chosen in accordance with Thompson et al.^12^. For large channel aspect ratios and low Reynolds number, upon applying a lubrication approximation and depth-averaging, the Navier–Stokes equations reduce to a single equation in terms of the non-dimensional fluid pressure *p*,

2 $$\begin{aligned} {\varvec{\nabla }}\cdot \big [ (b(y)^3 \, {\varvec{\nabla }}{p} \big ]=0. \end{aligned}$$

We impose no-penetration conditions on the channel side-walls, i.e. $p_y=0$ at $y= \pm \, 1$. At the bubble interface, we impose both a kinematic and dynamic boundary condition. We work in a co-moving frame of reference which translates at velocity ${{\varvec{U}}}(t) = (U_b(t), \, 0)$. Here, $U_b(t)$ is the speed of the bubble’s centroid in the streamwise direction and is obtained by requiring that the streamwise component of its centroid remains fixed at zero. The kinematic condition at the interface is then given by

3 $$\begin{aligned} \frac{\partial \mathbf{R} }{\partial t} \cdot \hat{\mathbf{n }} = \big [ -b(y)^2 \, {\varvec{\nabla }}{p} - {{\varvec{U}}}(t) \big ] \cdot \hat{\mathbf{n }}, \end{aligned}$$

where $\mathbf{R}$ denotes a point on the interface and $\hat{\mathbf{n }}$ is the associated outward-pointing unit normal vector. It is noted that all time-dependence is contained within Eq. (3). The dynamic condition at the interface reduces to the Young–Laplace equation. The in-plane curvature of the interface is denoted by $\kappa$. In the transverse direction, we assume that the bubble fills the entirety of the channel height, i.e. neglecting the presence of wetting films, and that the bubble is semi-circular with radius *b*(*y*)/2. Hence, the dynamic condition is given by

4 $$\begin{aligned} \llbracket p \rrbracket ^{\text {bubble}}_{\text {fluid}} = \frac{1}{3 \alpha Q} \bigg [ \frac{\kappa }{\alpha } + \frac{1}{b(y)} \bigg ], \end{aligned}$$

where the internal pressure of the bubble is obtained by requiring that the dimensionless volume of the bubble remains fixed to its initial value. The injection of fluid into the channel at constant flux is modelled by imposing a favourable pressure gradient in the streamwise direction, chosen such that the resulting dimensionless volume flux is equal to *Q*. The resulting system was then discretised and solved using a combination of finite-element and parameter-continuation methods contained within the open-source finite-element library oomph-lib^37^.

We impose systematic linear perturbations to a steady solution of the governing equations by applying the same eigenmode perturbation method described comprehensively in Keeler et al.^10^. Here, we will recall the important details of this method. We denote an arbitrary state of the system by the high-dimensional state variable *u*, which contains all of the problem unknowns, with associated time-derivative $\dot{u}$. The system of equations that we solve can be written in residual form as

5 $$\begin{aligned} \mathcal {R}(\mathbf{u} , \dot{\mathbf{u }}) \equiv \mathcal {M}(\dot{\mathbf{u }}) + \mathcal {F}(\mathbf{u} ) = 0, \end{aligned}$$

where $\mathcal {R}$ is a nonlinear operator that acts on the state variable and its time-derivative. It is assumed that $\mathcal {R}$ can be separated into two terms, consisting of a linear mass operator $\mathcal {M}$ that acts only on the time-derivative of the state variable and a nonlinear operator $\mathcal {F}$ that acts only on the state variable. Denoting a steady solution of Eq. (5) by $\mathbf{u} _s$, we form a perturbed state $\mathbf{u} _p$

6 $$\begin{aligned} \mathbf{u} _p = \mathbf{u} _s + \delta \mathbf{v} e^{\lambda t}, \end{aligned}$$

where $\vert \delta \vert \ll 1$ is a small perturbation parameter, **v** is a spatially-dependent eigenmode of the system and $\lambda$ is the corresponding growth rate of the perturbation. Insertion of the perturbed state into Eq. (5) and the application of a Taylor expansion about $\mathbf{u} _s$ yields

7 $$\begin{aligned} \lambda \mathcal {M} (\mathbf{u} _s) \mathbf{v} + \mathcal {J} (\mathbf{u} _s) \mathbf{v} = 0, \end{aligned}$$

at first-order in $\delta$, where $\mathcal {J}$ is a Jacobian operator. This is a generalised eigenvalue problem, which we solve numerically using the Trilinos library^38^ in order to determine the set of complex eigenmodes $\mathbf{v}$ and corresponding eigenvalues $\lambda$. An initial-value problem (IVP) is formulated using the least-stable eigenmode $\hat{\mathbf{v }}$

8 $$\begin{aligned} u(t=0) = \mathbf{u} _s + \varepsilon \hat{\mathbf{v }} e^{\text {i}\varphi } + \text {c.c.} \end{aligned}$$

where the strength and phase of the perturbation are controlled by the parameters $\varepsilon$ and $\varphi$ respectively. Depending on the values of these parameters, different initial perturbations are able to be imposed onto a bubble. The resulting unsteady time-evolution of the bubble was simulated using the implicit BDF2 timestepper. The time-step was chosen as $\Delta t=0.01$ and the validity of the solution was confirmed through a series of convergence tests carried out at each time-step. Simulations were terminated either when the bubble reached a state of steady propagation or broke up, the latter of which is indicated by the self-intersection of its boundary. The dynamics of multiple interacting bubbles are non-trivial and are not pursued in this paper. Instead, a comprehensive study can be found in Keeler et al.^32^.

## Supplementary Information

Supplementary Information.

## Data Availability

The datasets used and/or analysed during the current study are available from the corresponding author on reasonable request.
