# Supplementary material for: The unpredictable nature of bubble evolution
Source: Sci Rep. 2022 Dec 1;12:20752. doi: 10.1038/s41598-022-23231-8 (PMC9715726; doi:10.1038/s41598-022-23231-8)
Supplement: Supplementary file 1 — Supplementary Information. [file 41598_2022_23231_MOESM1_ESM.pdf]

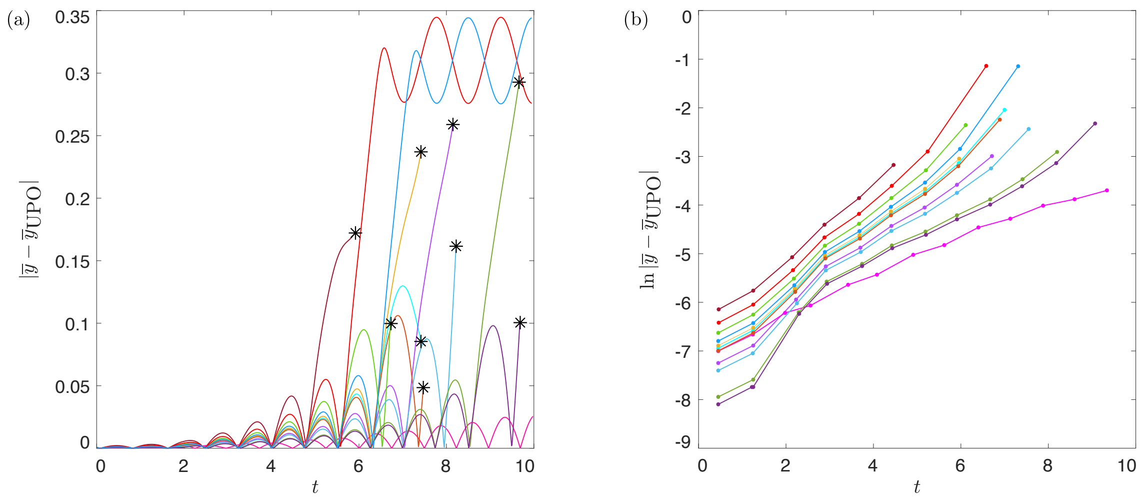

Figure S1: (a) Time-evolutions of the quantity $|\overline{y}-\overline{y}_{\mathrm{UPO}}|$, where $\overline{y}_{\mathrm{UPO}}$ is the lateral centroid offset of a bubble that remains on the UPO, for each of the simulated bubbles contained in Fig. 5 of the manuscript. The set of localised peaks of this quantity prior to either reaching a steady state or breakup are used to define a metric of separation from the UPO in (b).

In Fig. 1(a), we plot time-evolutions of the quantity $|\overline{y}-\overline{y}_{\mathrm{UPO}}|$, where $\overline{y}_{\mathrm{UPO}}$ is the lateral centroid offset of a bubble that remains on the UPO, for each of the simulated bubbles shown in Fig. 5 of the manuscript. The set of localised peaks of this quantity prior to reaching a steady state or breakup are used to define a metric of separation of trajectories from the UPO. The time-evolution of the logarithm of this metric is plotted in Fig. 2(a) for each of the bubbles. The metric increases approximately linearly in each case, indicating that the bubbles are diverging exponentially.

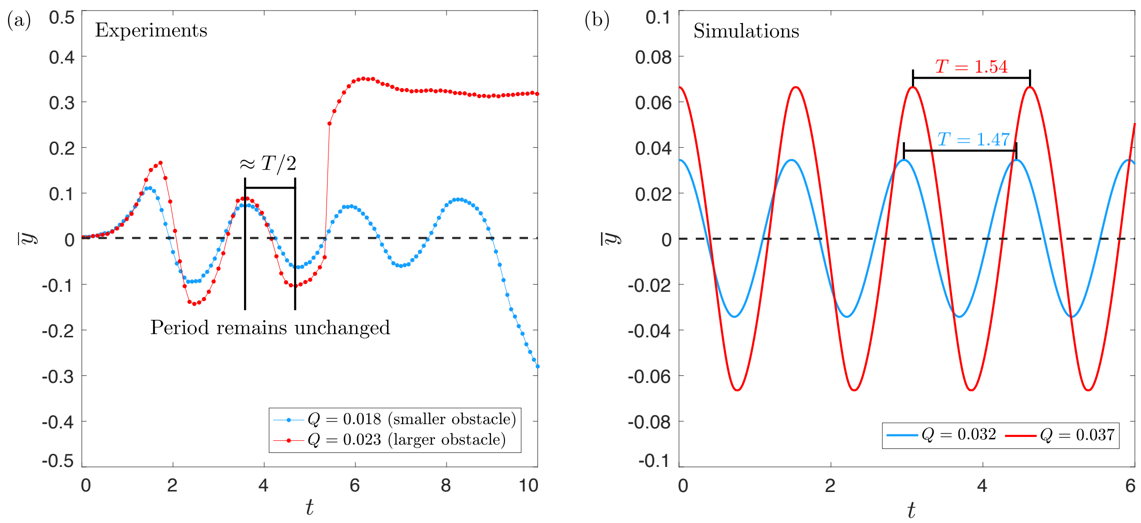

Figure S2: (a) Time-evolutions of the bubble centroid lateral offset, $\overline{y}$, for two bubbles that are perturbed by the smaller (blue) and larger (red) obstacles. In each case, the flow rate lies within the identified region of unpredictability. (b) Time-evolutions of $\overline{y}$ for two bubbles exploring the UPO in simulations at $Q = 0.032$ (blue) and $Q = 0.037$ (red).

In figure 2(a), we plot the time-evolution of $\overline{y}$ for two bubbles that are perturbed by the smaller (blue) and larger (red) obstacles within the identified regions of unpredictability. The interval of time between the second peak and trough are a reasonable estimate of the experimental UPO's half-period; this remains essentially unchanged upon increasing the flow rate, indicating that the UPO has not bifurcated and has merely increased in amplitude. Similar data is provided in figure 2(b) from the simulations. The computed period of the UPO, denoted T, remains essentially unchanged upon increasing the flow rate.
